# Survival among 148 patients with an incidentally detected appendiceal tumours at surgery for acute appendicitis: a population-based cohort follow-up study

**DOI:** 10.1007/s00068-024-02580-1

**Published:** 2024-07-17

**Authors:** Lennart Boström, Viktor Jovic, Martin Dahlberg, Fredrik Holtenius, Gabriel Sandblom, Hans Järnbert-Pettersson

**Affiliations:** 1 Department of Surgery, South General Hospital (Södersjukhuset), Sjukhusbacken 10, Stockholm, SE-11883 Sweden; 2 Department of Clinical Science and Education, Karolinska Institutet, South General Hospital, Stockholm, Sweden; 3 Department of Pathology, Karolinska University Hospital, Karolinska Institutet, Stockholm, Sweden

**Keywords:** Appendiceal tumour, Frequency, Acute appendicitis, Survival

## Abstract

**Purpose:**

To investigate the long-term prognosis of appendiceal tumours incidentally detected at appendicectomy for suspicion of benign appendicitis.

**Methods:**

A retrospective register-based single centre cohort study was carried out, using data from the local acute appendicectomy quality register of cases operated on at the Department of Surgery, South General Hospital, Stockholm, Sweden. The local colorectal cancer register was also used to identify appendix tumours. The study period was between January 2004 and January 2023. Survival was calculated according to the Kaplan-Meier method.

**Results:**

A total of 11,888 patients were registered in the acute acute appendicectomy register, 54% males and 46% females, median age 32 (Q1 = 21, Q3 = 47) (with 33.7% were 41 years or older). From the appendicectomy and colorectal registers 148 (1.2% of the total cohort) appendiceal tumours were found; 60% in females and 40% in males, median age 56 (Q1 = 43, Q3 = 70) (with 78.4% being 41 years or older). Tumours found were: Low grade Appendiceal Mucinous Neoplasms (LAMN, *N* = 64); Neuroendocrine Tumours (NET *N* = 24); adenocarcinomas or other form of carcinomas (*N* = 57); and adenomas (*N* = 3). The overall 5-year survival in patients operated for LAMN was 96.8%, for NET 93.3% and for adenocarcinoma 69.7%. The overall 5-year survival for all tumour patients was 85.7%. For the younger patients (< 51 years) with LAMN and NET, almost all survived to the end of follow-up. Survival of patients in the carcinoma group was statistically significantly lower than for the LAMN and NET groups, especially in females 51 years or older. In the group of tumour patients undergoing surgery (*n* = 146), primary surgery was laparoscopic in 47% and open in 52%. Two patients did not undergo surgery due to widespread disease. In 64% of cases operation was acute, whereas it was delayed and/or planned in 34%. Most procedures were laparoscopic appendicectomy 36%, followed by open appendicectomy 30%, right-sided hemicolectomy 14.6% (open 11.6% and laparoscopic 3%, acute operation 5.5%), ileocaecal resection 5% (acute operation 3.4%), and staging laparoscopy 7%. In 38% of the operated patients the tumour was discovered incidentally at histopathology examination. Two patients had CRS and HIPEC as the initial operation. Forthy-three per cent of the 146 tumour patients operated underwent a second procedure: CRS and HIPEC in 23.3% and right-sided hemicolectomy in 13.6% (laparoscopic 8.2% open 5.4%).

**Conclusion:**

Survival was high for patients with incidentally detected appendiceal LAMN or NET, but not so for carcinoma. Survival was lower in the carcinoma group older than 50 years, especially those sick and females.

## Introduction

Acute appendicitis (AA) is one of the most common conditions requiring acute surgery. Subsequent histopathology reveals that 0.5–2% of all appendices have some form of neoplasia [1–4]. The figure is higher with complicated AA [5]. Patients with conservative management of AA have an increased short- and long-term rates of bowel cancer [6]. The most common findings are LAMN (low-grade appendiceal mucinous neoplasm or mucocele), NET (neuroendocrine tumour or carcinoid), and appendiceal carcinoma including goblet cell carcinoma, and adenocarcinoma that was mucinous or with signet cell differentiation [7–13].

Perforation of the appendix due to LAMN can result in pseudomyxoma peritonei (PMP) with mucinous ascites and widespread peritoneal growth. Adenocarcinoma in the appendix may cause peritoneal and lymph gland metastases, as well as hematogenous spread.

The aim of the current study was to analyse the rate of incidentally discovered appendiceal tumours, type of tumours, relation to age and sex, form of surgery, and finally long-term survival.

## Methods

Patients undergoing appendicectomy 1st January 2004–31st January, 2023 at South General Hospital were eligible for inclusion. The last day of follow-up for death and survival was May 15th, 2023.

Since 2004 the Department of Surgery, South Stockholm General Hospital, Sweden, has had a prospectively recorded quality register with information on all patients undergoing appendicectomy for AA [14]. South General Hospital, Stockholm, provides emergency medical care to a geographically well-defined catchment area of approximately 700 000 inhabitants. The patients operated are continuously identified through the local operation logistic software (Orbit, Tieto Evry, Kristianstad, Sweden, the Orbit 4 edition; January 2004- November 2015, and the Orbit 5 edition; December 2015– January 2023). Procedure-related data are transferred to the register from Orbit, and other parameters from electronic medical records (Melior, Siemens Health Services) from January 1, 2004– October 31, 2011, and Take Care (Compu Group Medical, Helsinki, Finland) from November 1, 2011– January 31, 2023.

Prior to September 2016, only AA patients aged > 14 years were treated at the Department of Surgery at Stockholm South General Hospital. Younger patients were operated at the Karolinska University Hospital, by paediatric surgeons. However, since September 2016, all paediatric AA patients aged 10–14 years are now treated at Stockholm South General Hospital. These patients are admitted to a pediatric ward, but general surgeons take medical decisions and go daily rounds.

Our quality register for colorectal surgery (KVALOG) was started in 2006 and includes data from Melior and later from Take Care. From this register acute and electively operated appendiceal tumours, not included in the appendicitis register, could be identified. Acute operations for AA where an appendiceal tumour was found are noted in the local AA Register. Some patients with an appendiceal tumour can be included in both registers.

All patients admitted with suspected appendicitis at the Department of Surgery at South General Hospital are managed according to a standard regime. Open appendicectomy for AA was most common during the early part of the study period but was gradually replaced by laparoscopic appendicectomy. All specimens were examined macroscopically, and histopathology examination was done selectively during the first years and routinely during final years of the study. If suspicion of a tumour of the appendix was raised intraoperatively and the proximal part of the appendix seemed uninvolved, then appendicectomy was considered an option. In case of uncertainty regarding tumour growth right-sided hemicolectomy, or ileocecal resection was preferred. Appendicectomy with planned reoperation after histopathology examination and a multidisciplinary conference was also an alternative strategy. In case of uncertain radicality of the tumour extended resection was sometimes carried out. Patients with a pre- or intraoperative suspicion of a tumour in the appendix, without the need for acute surgery, often underwent planned surgery with high priority within a week or so. In the case of peritoneal carcinosis or pseudomyxoma peritonei (PMP) cytoreductive surgery (CRS) and hyperthermic intraperitoneal chemotherapy (HIPEC) [15–16] was considered. This treatment was carried out at the Department of Surgery, Uppsala University Hospital, Sweden from 2005 to 2012, and at the Karolinska University Hospital, Stockholm, Sweden 2012–2023. The operation included right-sided hemicolectomy, resection of the omentum majus, peritonectomy, hysterectomy and bilateral oophorectomy, and resection of other organs affected by tumour, such as resection of small intestine and colon. The procedure was carried out with the intention to excise all tumour growths in the abdominal cavity. Finally, cytostatic drugs were instilled in the abdominal cavity.

### Pathological-anatomical diagnosis (PAD)

Categorisation of tumours was made using the 5th Edition 2019/TNM8 World Health Organisation (WHO), Classification of tumors of the digestive system was used [17].

In the early part of the study period, macroscopic examination was routine intraoperatively while histopathologic examination was done selectively in cases with macroscopic suspicion of tumour.

### Statistical analyses

Main outcome was long-term survival and death. Last day for follow-up for death was May 15th, 2023. The Kaplan-Meier method was used to estimate survival time after operation. Time at risk was calculated as time from operation to death (event) or censored at the end of the study (May 15th, 2023), whichever occurred first. Differences in survival between groups (LAMN, NET, and cancer) were assessed using log-rank-test.

Only patients with primary tumour in the appendix were included in the analyses. Patients with a secondary tumour in the appendix from another organ were excluded.

A *p*-value (two-sided) of 0.05 or less was considered to indicate statistical significance.

## Results

### Gender and age

During the period January 2004 to January 2023 11,888 appendicectomies were performed due to suspicion of AA at South General Hospital in Stockholm; 46% were females and 54% males. From the combined AA and colon cancer registers, 148 patients with an appendix tumour were found, 89 females (60%) and 59 males (40%), all except two (with widespread disease) were resected. Of these patients 107 came from the AA register and 97 from the KVALOG, i.e. some were recorded in both registers. Age groups for patients with AA, appendix tumor and rate of tumours compared to AA are presented in Table 1.

**Table 1:** Numbers of operations for AA and appendiceal tumours in age-groups

Age-groups	Acute appendicitis		Tumour-patients		Tumors/AA
	No.	%	No.		%
<_ 18	2455	20.7	4	2.7	0.2
19–30	3047	25.6	8	5.4	0.3
31–40	2381	20.0	20	13.5	0.8
41–50	1521	12.8	22	14.9	1.4
51–60	1179	9.9	32	21.6	2.7
61–70	785	6.6	26	17.6	3.3
71–80	382	3.2	27	18.2	7.1
81–90	122	1.0	8	5.4	6.6
91+	15	0.1	1	0.7	6.7
total	11887	100	148	100	
	missing 1		2 not op		
	Acute appendicitis		Tumour patients		
Gender		%	No.	%	
females		46.3	89	61.0	
males		53.7	59	39.0	
Op 2004–2011		33.1	53	36.3	
Op 2012–2023		66.9	93	63.7	
Histopathology	No.	%	No.	%	
2004–2011	1665	43.3	53	100.0	
2012–2023	6149	79.2	93	100.0	
Radiology, CT/US					
2004–2011	2072	53.9			
2012–2023	7466	96.1			

For the group with a tumour the median age were 56 years (Q1 = 43, Q3 = 70) (78.4% were 41 years or older), while the corresponding figure for the patients with AA were median age 32 (Q1 = 21, Q3 = 47) (33.7% 41 years or older) (Table 1).

The use of preoperative imaging, abdominal ultrasound (US) or computer tomography (CT), was 30% in 2004 increasing to 93% in 2014 [14]. In 2022, 99% of patients had a preoperative radiology.

### Mortality and survival in tumour patients

Among the 11,888 patients in the AA register 14 (0.12%) patients died within 30 days after the operation. These were elderly patients with multiple comorbidities. By the end of the follow-up, 35 tumour patients had died, 19 women and 16 men.

**Fig. 1:**
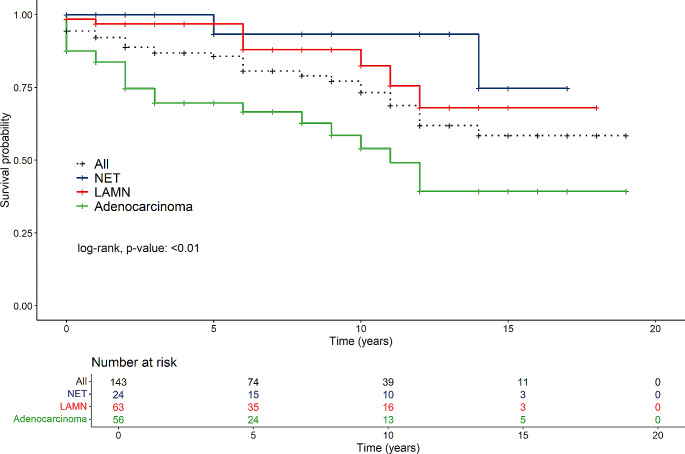
Survival of all tumour patients in the NET-, LAMN- and adenocarcinoma groups

The overall 5-year survival in patients operated for LAMN was 96.8%, for NET 93.3% and for adenocarcinoma 69.7% (Fig. 1). The overall 5-year survival for all tumour patients was 85.7%.

In the tumour group 76% were still alive at the final day of follow-up. One-year survival was 89.2%, 5.4% died and 5.5% were not followed-up one year after the operation. Five-year survival was 51.4%, 13.7% died and 34.9% still alive were not followed up 5 years after the operation.

Most of patients survived one year, but many had not been followed up at 5 years postoperatively by the last follow-up date. The majority of those who died were sick and elderly.

Patients who received adjuvant treatment had a poorer 5-year survival; 56% compared to 90% alive without adjuvant treatment.

**Fig. 2:**
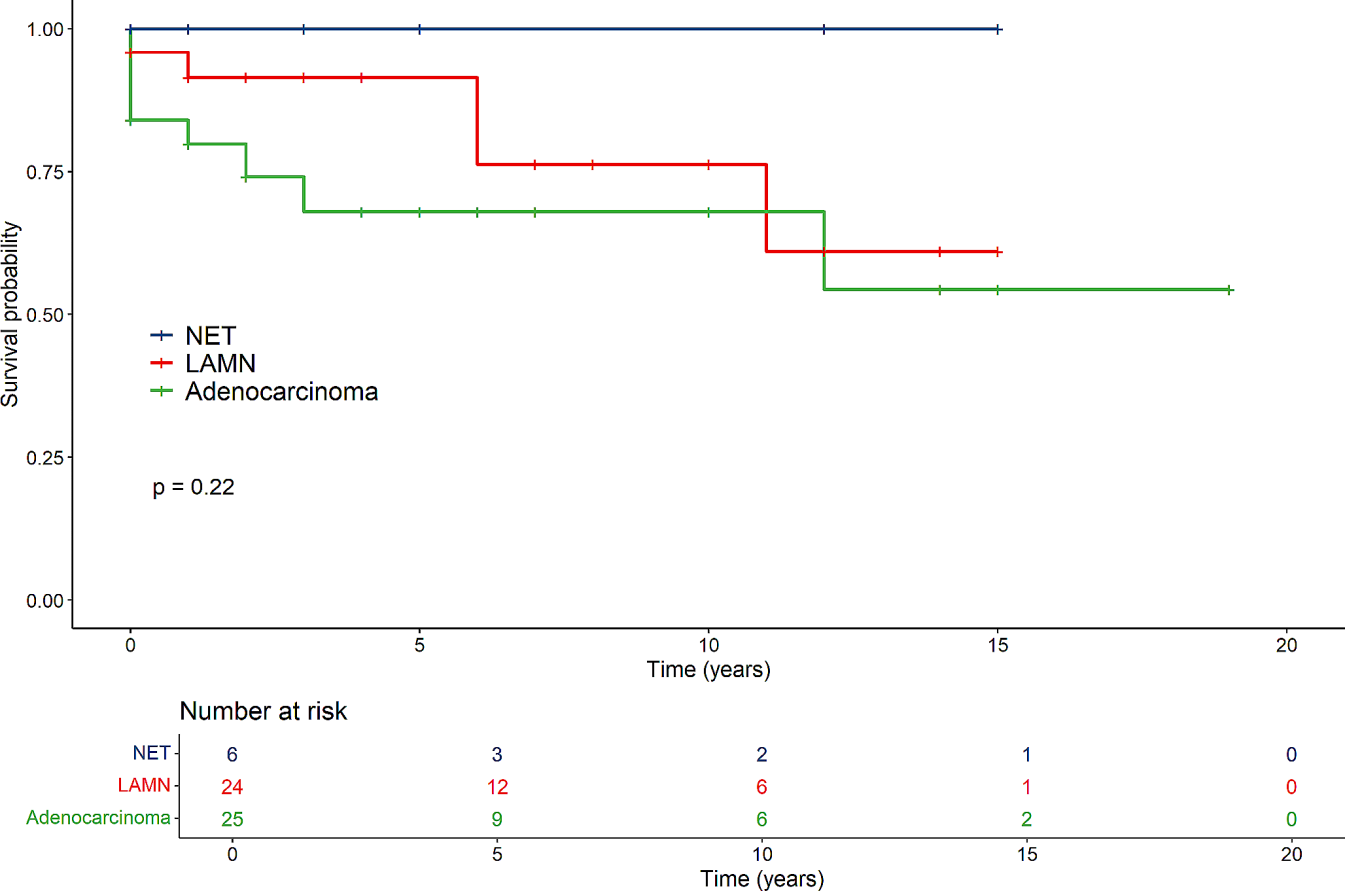
Survival of females in the NET-, LAMN- and adenocarcinoma groups

**Fig. 3:**
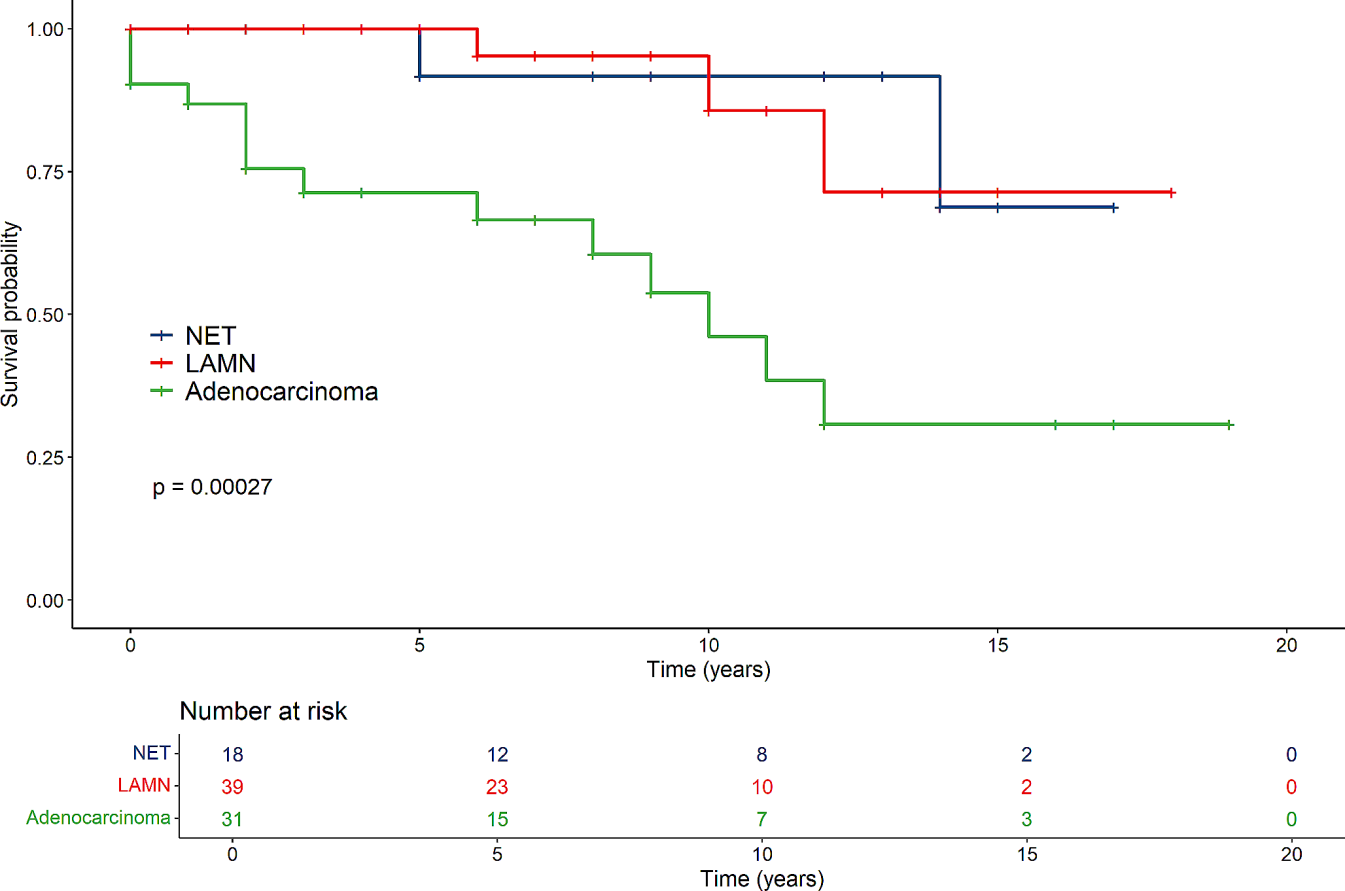
Survival of males in the NET-, LAMN- and adenocarcinoma groups

In general, groups differed regarding survival time (NET, LAMN and Carcinoma), where the Carcinoma-group had the lowest survival time (*p* < 0.001) (Fig. 1). Females in the cancer group had lower survival compared to those in the LAMN- and NET-groups (*p* < 0.001) (Fig. 2). For males there were no difference between the tumour groups (*p* = 0.22) (Fig. 3).

**Fig. 4:**
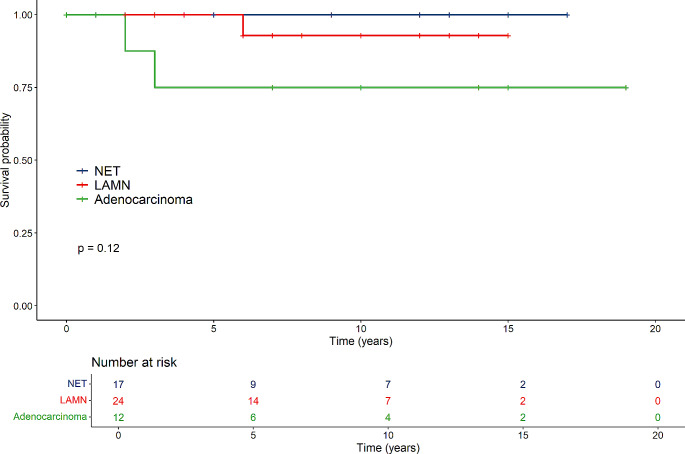
Survival of patients older than 50 years in the NET-, LAMN- and adenocarcinoma groups

**Fig. 5:**
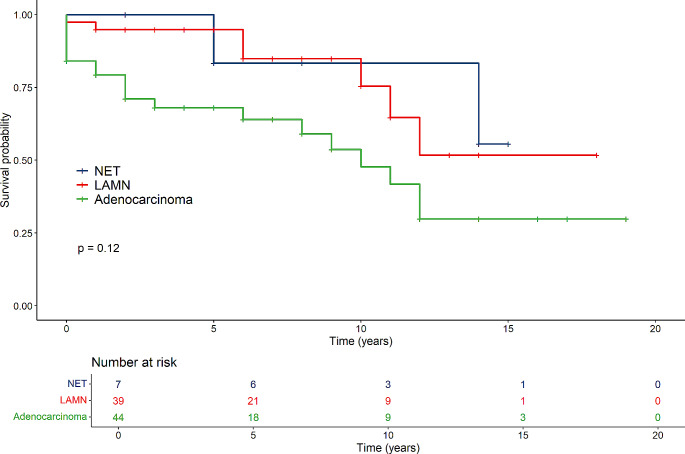
Survival of patients 50 years or younger in the NET-, LAMN- and adenocarcinoma groups

Patients younger than 50 years at surgery had similar survival rates in the three groups (*p* = 0.12), but the number of patients was small (Fig. 4**).** No differences in survival were seen between the tumour groups among patients older than 50 years at the time of operation (Fig. 5).

### Surgery for appendix tumours

At surgery, 26.2% of the AA patients had a perforation. The perforation rate did not change significantly over the period of the study. Tumour patients had approximately the same perforation rate (25.3%) as patients with benign acute appendicitis.

The surgeon suspected a tumour intraoperatively in 62% of the tumor patients, whereas in 38% the tumour was discovered incidentally at histopathology examination. Of those who were diagnosed with appendiceal tumours, suspicion was already raised at preoperative radiology in 41% of cases.

In the AA quality register during the study period, 62.5% underwent laparoscopic appendicectomy, 33% appendicectomy through an open transverse incision, 1.9% through an open midline incision and 0.1% through an open paramedian incision. In 2004, 6% were laparoscopic appendicectomies and in 2022 the figure was 99%.

In the group of tumour patients undergoing surgery (*n* = 146) primary surgery was laparoscopic in 47% and open in 52%. Two patients did not undergo surgery due to widespread disease. In 64% of cases operation was acute, whereas it was delayed and/or planned in 34%. Most procedures were laparoscopic appendicectomy 36%, followed by open appendicectomy 30%, right-sided hemicolectomy 14.6% (open 11.6% and laparoscopic 3%, acute operation 5.5%), ileocecal resection 5% (acute operation 3.4%) and staging laparoscopy 7%. Two patients had CRS and HIPEC as the initial operation.

Forty-three per cent of the 146 tumour patients operated underwent a second procedure. CRS and HIPEC in 23.3%, and right-sided hemicolectomy in 13.6% (laparoscopic 8.2%, open 5.4%).

At primary surgery, the surgeon did not suspect a tumour in 38% of the cases, but a tumour was later found at histopathology. In the remaining there was at least a minor suspicion of tumour (62%). When the primary surgery was performed, a radical resection of the tumor was seen in 65% of the 146 operated tumour patients. Of 148 patients, 107 (72.3%) were followed up in the policlinic and 41 (27.7%) were not.

### Postoperative complications in tumor patients

93% of the tumour patients had no surgical complication after the primary procedure. Two had intraabdominal abscesses, 8 paralytic ileus and 4 surgical site infection. Ten patients suffered various medical complications.

### Tumour patients and PAD

In 2004, only 36.6% of the appendicectomies carried out for benign AA had a specimen sent for histopathology examination (PAD), while in 2022 the rate was 96.3%. All tumours in the present study were confirmed at histopathology.

Among the 148 patients with an appendix tumours; 64 had LAMN, 57 carcinomas of some form, 24 NET and 3 an adenoma. Of those with LAMN, 15 had pseudomyxoma peritonei at the time of diagnosis. The carcinoma group included: adenocarcinoma, signet ring adenocarcinoma, goblet cell carcinoma and mucinous adenocarcinoma. In this group 8 had peritoneal carcinosis at diagnosis. Two did not undergo appendicectomy since the tumour was found to be disseminated at diagnosis (radiology and biopsy).

### Follow-up, oncology treatment, recurrence

Of the tumour patients 28% went without follow-up, whereas 72% were followed at least with at least outpatient visits. The majority of those who were followed up did not undergo adjuvant treatment (64%), whereas 34% received adjuvant therapy of some kind. At the final day of follow-up May 15th, 2023, 79% of the tumour patients were without recurrence, 10% had disseminated cancer already at diagnosis, and 11,5% developed recurrent disease.

### Hospital stays after the primary operation

53% of the tumour patients stayed 1–3 days after the first or only operation, 26% 4–6 days and 21% more than 6 days.

### Causes of death in tumor patients

More than half(18) of 35 patients who died had disseminated cancer as cause of death, especially appendix/colorectal cancer (51.4%), the remainder died of causes not related to the appendix cancer. Of those who died, 32 were 51 years or older at the operation. Cardiac infarction, stroke, pneumonia and hepatic failure were the first obvious causes of death in several cases, while the cause of death was unknown in 3 cases.

## Discussion

In the present population-based cohort of patients undergoing appendicectomy for assumed benign appendicitis, survival of those with an incidentally detected appendiceal tumour was favorable. Nevertheless, the patients with carcinoma had a poorer prognosis than those with NET or LAMN and we recommend that these be paid special attention to when deciding on follow-up. The rate of occult appendix tumours during the period studied was 1.2% which is in line with other reports [1–4, 18].

In the beginning of the study period (2004) 70% of patients in the AA register did not undergo preoperative radiology. The use of radiology gradually increased, and at the end of the study period, 99% of the patients had US or CT scan of the abdomen prior to surgery. The increase in preoperative radiology increases the diagnosis rate of tumour leading to delayed or planned surgery instead of acute appendicectomy. But small tumours of the appendix are difficult to detect with US and CT scan and might have been missed in this patient material.

Histopathology was performed in about 1/3 of the appendicectomy in the beginning of the study period, increasing to about 96% at the end. There is no way of knowing how many appendix tumours remained undetected during the study period. However, those not seen macroscopically will probably have been small and radically excised. There was no suspicion of appendix tumour at surgery in 38% but was a surprising find at the histopathology. Fortunately, 2/3 of the tumour patients had a radical operation performed at the first operation.

The one-year survival was very high (89%) compared to tumours in the upper gastrointestinal tract. The five-year survival was also high, but there were some deaths among older patients with an overweight for women. In the NET and LAMN groups the long-term survival rate was high. In the carcinoma group, patients older than 50 years, especially women, had a poor survival rate compared to the LAMN and NET groups. This has also been confirmed in a previous report [19]. Skendelas et al. also found that appendiceal adenocarcinoma was associated with older age at the time of surgery for acute appendicitis [20].

All patients receiving postoperative adjuvant chemotherapy treatment survived one year after resection. Of the 39 patients with adjuvant treatment surviving 5 years, 72% survived to the end of follow-up.

Hospital stay after the first operation was short, and most patients were discharged within a week.

Among the causes of death, 51% of the patients had appendix/colorectal cancer disease as cause of death, especially those with spread appendix cancer at time of diagnosis.

In the tumour group, 66% underwent open or laparoscopic appendicectomy. Colonic resections predominated among those undergoing a second procedure. Also, palliative non-curative surgery was performed in patients with severe disseminated cancer disease. Among the 148 patients with appendix tumour, about 15% had pseudomyxoma peritonei or peritoneal carcinosis at the first operation. With advanced secondary treatment, the survival of these patients has increased [16]. Total CRS with HIPEC was the most common procedure for patients with PMP or peritoneal carcinomatosis [15, 16]; a treatment that increases long-term survival [21].

Guzman et al. [22] found no survival advantage when performing colectomy after appendicectomy in a large cohort of patients with a non-metastatic carcinoid tumour of the appendix (NET). In our department, decisions on further surgery and chemotherapy are made at a multi-disciplinary team conference. Further research is needed before we can change recommendations for management of appendiceal carcinoid tumours.

In a nationwide study on children with an appendiceal tumours Parikh et al. concluded that appendicectomy may be sufficient and that there is no need for extensive surgery [23]. Extensive resection may not increase patient survival but puts the patient at greater risk. They found no significant difference in 15-year survival between appendicectomy vs. extensive resection (the tumours were carcinoid 72%, adenocarcinoma 16% and lymphoma 12%).

For goblet cell tumour (GCT) of the appendix, a prophylactic right-sided hemicolectomy is recommended to reduce the risk for recurrence [24]. Overall 5-year survival rate was 41.6% for patients with a GCT. GCTs are more like colorectal adenocarcinomas than to NETs, and systemic and adjuvant chemotherapy is advised for these tumours. In a previous study the overall 5-year survival was approximately 60% for patients with appendix adenocarcinoma [25].

In a study of 612 patients with LAMN Köhler et al. reported an overall 5-year survival rate of 79.5%. If the patients were younger than 55 years at the time of diagnosis the survival rate was 85.8%, i.e. significantly higher [26]. This was confirmed in another study by Sugarbaker et al., where LAMN had a mean survival of 24.5 years [27].

### Strengths and limitations

A strength of this single centre study is the size of the cohort comprising almost 12,000 patients undergoing appendicectomy. Another strength within is that gathered data from our own AA and KVALOG quality registers. The Stockholm South General Hospital has a well-defined catchment area, the study group thus represents the entire population covered by the hospital apart from those seeking healthcare while on a temporary visit to the catchment area. The personal identification numbers in Sweden makes it possible to trace all residents regardless of migration or change of health care provider. This has made it possible to follow each patient during the entire period of the study. A limitation is the low number of tumour patients and that this was a retrospective study. There may also have been patients with a localised tumour that was missed at macroscopic inspection and were no specimen was sent for histopathology, at least during the first years of the study. This could have led to selection bias.

## Conclusion

In the present cohort of patients undergoing acute appendicectomy for suspected benign acute appendicitis, an appendiceal tumour was found in 1.2% of specimens. Of these 25% were perforated. There was no suspicion of tumour preoperatively in 61% of the patients. During the operation the surgeon was unsure about the diagnosis in 38% of cases. The primary operation was radical in 2/3 of tumour cases. Over the period of the study open appendicectomy was gradually replaced by laparoscopic appendicectomy. When necessary, a second more extensive operation should be considered to make sure no tumour remains in the abdomen. In case of dissemination in the abdominal cavity CRS and HIPEC may be indicated. Patient survival is high for LAMN and NET but is lower for appendiceal adenocarcinomas, especially in older patients and women.

## Data Availability

No datasets were generated or analysed during the current study.
